# Payment for Military Patients and Treatment of Discharged Warriors

**Published:** 1917-12-08

**Authors:** 


### THE LONDON HOSPITAL.

### Payment for Military Patients and Treatment of Discharged Warriors

At the Quarterly Court of the Governors, held on December 5, the House Committee's Report for the past quarter was read.

Air Raids.

Since the last Court, the Hospital has had further experience of air raids. The Committee have modified their former decision, and have agreed to give shelter during raids to 2,000 women and children in the basements. It is not altogether clear whether it is the duty of a hospital to provide this shelter. Its first duty is to the patients lying helpless in its beds, and to be ready to give treatment to injured people who may be brought to its doors as the result of a raid. The crowds in the basement occupy the attention of some of the staff who ought to be free to be in the wards with the patients, or to be in the receiving room; the filth that is brought in and left behind is not good for the hospital, and more wages have to be paid to the night scrubbers for the extra work entailed; the presence of these refugees throws much extra work on a staff already overworked and overstrained. Once inside, the behaviour of the crowd has been orderly, but the crushing at the entrance, largely due to hundreds of young fellows who apparently are healthy, strong, and of military age, is dangerous to the women and children, while the latter appear to have been used as a sort of ticket of admission by some of the men. The Committee have decided, therefore, to admit only women and children up to 2,000, and no more, and this has been done with the entire agreement of the police, who state that there is ample cover in the district for 152,000 people. Men must in future go elsewhere.

East End Emergency Maternity Wards.

Scores of applications have been received from women soon to be confined to be admitted to the beds of the hospital during the period of raids. The hospital, cannot, however, admit more than it is doing, and has already put up extra beds in the lying-in wards. Application was made to the Local Government Board for help and advice. It has been arranged that a certain part of Whitechapel Infirmary shall be, as it were, lifted out of the infirmary, and this part shall be known as the East End Emergency Maternity Wards. It is not a pauper establishment at all, and no name of a patient entering will appear in any Poor-Law book or record. There are forty beds, and these are under the charge of the Chief Medical Officer, Dr. Woodyatt, always helpful, and his assistants, with four qualified midwives. The admission to these beds is subject to Dr. Woodyatt's entire liberty of discretion, and will be arranged by the maternity almoner of the London Hospital, who will weekly supply Dr. Woodyatt with a list of women personally known to her, and will give to each woman a ticket which will admit her when taken to the infirmary at the time of need. The Committee returns very grateful thanks to Sir Arthur Downes for his prompt and invaluable help, to the Guardians of Whitechapel Infirmary, and to the staff of that excellently managed institution. There is no charge on the rates. The women are expected themselves to contribute towards the cost, and the difference will be made up from a certain charitable fund.

The Admission of Wounded.

The hospital continues to admit wounded—100 officers, 16 men suffering from spinal and head injuries, and 6 suffering from injuries to the eyes. A bed is rarely empty, and the officers' empty beds have to be notified morning and evening. The military portion was inspected by an official from the War Office quite recently, and highly commended for its work. The War Office pays the hospital 4s. a day for taking in wounded men. The London and other hospitals are trying to get this figure increased. If it was adequate at the beginning of the war—which it was not—it is far from sufficient now. As a matter of fact, every patient of the London costs nearly twice as much per day as this, although the surgeons and physicians give their services gratuitously.

Treatment of Discharged Sailors and Soldiers.

The treatment of discharged sailors and soldiers is receiving the Committee's attention. These cases are often likely to require a residence of long duration—electrical treatment, massage and exercises; there are cases of nervous breakdown vaguely spoken of as "shell-shock patients"; cases of deafness; of painful amputation stumps; cases suffering from a foreign body left in the body which has worked to a nerve and become painful—and so on. For the treatment of all cases where the illness has been caused or aggravated by the war the nation is responsible, and the Ministry of Pensions is authorised to make the necessary arrangements. The Government may arrange for special hospitals to be founded to treat these cases, or arrangements may be made with voluntary hospitals already in existence for the treatment of these cases under certain conditions. The Committee feels that these conditions are that—

1. The entire cost of the patient to the hospital should be defrayed by the nation, which is responsible.

2. The charge should include payment to the medical staff, who ought not to be asked to do this service gratuitously.

The Committee are now considering fully this question, and are in communication with other hospitals concerning it. The Court will see that it is one of great importance and some difficulty. Many interests are involved—first and foremost, that of the patient himself, that of the Government, that of the staffs of the voluntary hospitals, of the subscribers to the hospitals, and of the general practitioners outside.

The Committee's Gift to Mr. Foster.

At the meeting of the Committee on Monday last his colleagues presented Mr. Foster with a silver inkstand, engraved, to mark their appreciation of the services he has rendered the hospital during the war. He has filled the post of assistant secretary, has been at the hospital daily, with only a short summer holiday, and has slept at the hospital during the house governor's holidays. Mr. Foster has remained in residence every alternate week-end for the same reason. It would not have been possible for the administrative work to have been carried on in this time of stress but for his freely offered and gladly accepted help. No one has done more useful war work than Mr. Foster. Further, its chief merit, unlike that of much voluntary assistance, has been its regularity and persistence.

Items of Interest.

There will be no maternity students at the London until May next, the work meanwhile will be carried on by nurses who have obtained the Central Midwives Board Certificate; Dr. O'Donovan, on the staff of the Ministry of Munitions, and an old "Londoner," has been appointed examiner of probationers; Mr. Langton, formerly head of the Forget-Me-Not Bond Department, has been elected head of the Appeal Department; all departments that have to do with the collection of funds are being brought under one head; the hospital has received a cheque for £371, being a return by the Government on duty paid on alcohol.

Next year is the Quinquennial Appeal Year, and special efforts will be made to collect enough to meet the enormously increased expenditure of the hospital; the co-operation of every governor is most essential; there will be a deficit in this year's accounts of about £8,000—considering that the expenditure has increased to about £170,000, it is a matter of congratulation that the deficit is not greater.

